# Physical recovery modalities in competitive and elite endurance athletes: a systematic review and network meta-analysis finding limited evidence of benefit over passive recovery

**DOI:** 10.3389/fspor.2026.1876924

**Published:** 2026-08-18

**Authors:** Yu Jin, Dandan Pan, Mengying Li, Dinghuang Zhu, Jian Wen

**Affiliations:** 1 Chongqing Institute of Sports Science, Chongqing, China; 2 Shanghai Elite Sport Training Administrative Center, Shanghai, China; 3 College of Physical Education and Health Science, Chongqing Normal University, Chongqing, China; 4 School of Competitive Sports, Shanghai University of Sport, Shanghai, China; 5 Department of Pain and Rehabilitation Medicine, Second Affiliated Hospital of Army Medical University, Chongqing, China

**Keywords:** endurance athletes, exercise-induced muscle damage, network meta-analysis, physical recovery modalities, placebo, systematic review

## Abstract

**Background:**

Physical recovery modalities are routine in elite sport, yet whether any outperforms passive recovery, and whether the evidence supports modality-specific recommendations, remains unestablished.

**Methods:**

We searched PubMed, Embase, CENTRAL and Web of Science from inception to 1 April 2026 for randomised controlled trials of physical recovery modalities in competitive endurance athletes. Random-effects network meta-analyses were fitted for eight outcomes with passive recovery as reference, reporting standardised mean differences (SMDs) with 95% confidence intervals and *P*-scores. Certainty of evidence was rated with CINeMA against a minimal important SMD of 0.5, and risk of bias with RoB 2 (PROSPERO: CRD420261368621).

**Findings:**

Sixteen RCTs (273 participants) met inclusion; fourteen contributed to at least one network. Of 47 contrasts against passive recovery, three excluded the null, but none robustly: two rested on single-trial nodes and all three on networks pooling incommensurable measures. No modality outperformed passive recovery for delayed-onset muscle soreness (5 trials; *I*-squared 90.2%), creatine kinase (4 trials), or sport-specific performance (11 trials; *I*-squared 0%). The evidence base was thinner than the trial count implies: 35 of 55 nodes (64%) rested on a single trial, and restricting to confirmed elite participants left four of eight outcomes with no estimable network. A thermoneutral-immersion sham ranked at or above every active modality on all three outcomes. Certainty was very low for 31 of 47 comparisons and low for 15; the only moderate-certainty finding was that compression garments do not improve performance (SMD = 0.04, 95% CI −0.37 to 0.45).

**Interpretation:**

No modality has been shown to outperform passive recovery on any outcome examined. For sport-specific performance this is an informative null: intervals for compression garments, active recovery and electrical stimulation exclude clinically important effects in both directions. Elsewhere the randomised base is too small and heterogeneous to support the modality-specific recommendations routinely made—the muscle-soreness network reverses when two of its five trials are removed—and a sham performing as well as the active modalities indicates that expectancy cannot be separated from physiological effect in this unblinded literature. Adequately powered, sham-controlled trials are required before these modalities can be recommended on efficacy.

**Systematic Review Registration:**

PROSPERO CRD420261368621.

## 1 Introduction

Elite endurance sports are characterised by congested competition schedules and extreme physiological demands, requiring athletes to maintain peak performance under significant homeostatic stress (1, 2). In events such as the Olympic Omnium, Grand Tours in cycling, or multi-stage trail running, the recovery window between high-intensity bouts is often restricted to hours or even minutes (3, 4). Within this professional context, the rapid elimination of fatigue and the restoration of neuromuscular function are widely treated as decisive rather than supplementary factors in competitive success (5, 6).

The biological underpinnings of post-exercise performance impairment are multifactorial, involving metabolic acidosis, the accumulation of metabolic waste products such as blood lactate, and exercise-induced muscle damage (EIMD) (7, 8). Strenuous endurance efforts, particularly those involving substantial eccentric loading or high-intensity surges, lead to sarcolemma disruption and an inflammatory cascade, clinically manifested as delayed-onset muscle soreness (DOMS) and elevated circulating creatine kinase (CK) (9, 10).

A wide range of physical modalities is deployed against these processes. Active recovery is used to accelerate lactate clearance (11, 12); compression garments and intermittent sequential pneumatic compression (ISPC) are used to support venous return and limit swelling (13–15); cold-water immersion and whole-body cryotherapy are used to blunt the inflammatory response and reduce perceived soreness (16, 17); massage, neuromuscular electrical stimulation and far-infrared therapy are each advocated on distinct mechanistic grounds (18–20). These modalities differ by orders of magnitude in cost, staffing and logistical burden, and elite programmes commit substantial resources to them.

Two features of the evidence make comparative synthesis both necessary and difficult. First, most trials compare a single modality against passive rest, so the comparative question that practitioners actually face—which modality, for which purpose—has rarely been addressed directly. Network meta-analysis can in principle combine direct and indirect evidence to answer it. Second, elite athletes may possess faster innate recovery capacity than the recreational populations in which many recovery interventions were first tested, which would narrow the therapeutic window available to an external modality and make effects harder to detect (21, 22). An elite-specific synthesis therefore cannot simply inherit conclusions from the wider literature.

A third feature is less often acknowledged. Physical recovery modalities cannot be blinded: an athlete who has been massaged, or who has left a cryotherapy chamber, knows it. The outcomes most often reported—soreness and perceived exertion—are precisely those most responsive to expectation. Sham and attention-control conditions, which would allow expectancy and physiology to be separated, are rare in this literature.

We therefore conducted a systematic review and network meta-analysis to address three questions: (i) does any physical recovery modality outperform passive recovery in competitive and elite endurance athletes, across physiological, functional and perceptual outcomes; (ii) how much randomised evidence actually underpins each comparison; and (iii) can the observed effects be distinguished from expectancy?

## 2 Methods

### 2.1 Protocol and registration

The protocol for this systematic review and network meta-analysis (NMA) was prospectively registered with the International Prospective Register of Systematic Reviews (PROSPERO: CRD420261368621). The study was conducted and reported in accordance with the PRISMA-NMA extension statement. Deviations from the registered protocol arising during revision, including the re-coding of intervention arms described in Section 2.3 and the addition of a certainty-of-evidence assessment, have been recorded with PROSPERO.

### 2.2 Search strategy and eligibility criteria

A systematic search was performed across four electronic databases: PubMed, Embase, the Cochrane Central Register of Controlled Trials (CENTRAL), and Web of Science, from their inception through April 2026. No language restrictions were applied. The full search string for each database, with the date each search was run and the number of records retrieved, is provided in Supplementary Table S2. No RCT filter was applied in CENTRAL, which is already restricted to trials. The search combined Medical Subject Headings and free-text terms relating to the population, the interventions, and study design.

Studies were eligible if they met the following criteria. Participants: competitive, elite or professional endurance athletes (VO2max > 55 mL/kg/min for males and >50 mL/kg/min for females, or Tier 3–5 in the participant classification framework). Interventions: physical recovery modalities, including cold-water immersion (CWI), whole-body cryotherapy (WBC), active recovery (AR), manual massage, compression garments (CG), and neuromuscular electrical stimulation (NMES). Comparators: passive recovery, placebo or sham interventions, or head-to-head comparisons of active modalities. Outcomes: subjective muscle soreness (DOMS) and creatine kinase as primary outcomes; performance restoration, blood lactate clearance (BLA), rating of perceived exertion (RPE), interleukin-6, flexibility and countermovement jump or maximal voluntary contraction as secondary outcomes. Study design: randomised controlled or randomised crossover trials.

### 2.3 Study selection and data extraction

Literature management and screening were performed using Covidence. Two reviewers screened titles and abstracts, followed by full-text assessment, with discrepancies resolved by consensus or adjudication by a third reviewer. Data were extracted into a structured workbook capturing, for each trial arm, the intervention as described by the original authors, sample size, and arm-level means and standard deviations for every outcome.

During revision, and following a reviewer's observation that one trial had been misclassified, every arm of every included trial was re-coded directly from the intervention descriptions recorded in the extraction workbook and re-checked against the source publication. Four trials had been coded as delivering interventions they did not deliver, and these codes were corrected. The intervention coding used in the present analysis, together with the verbatim arm description from which each code was derived, is provided in Supplementary Table S8 so that the coding can be checked independently.

Where a trial contributed several doses of the same modality [three compression pressures in (23); two compression protocols in (24)], the arms were combined into a single group using the method of the Cochrane Handbook (Section 6.5.2.10) before analysis, consistent with treating the modality as one node. A sensitivity analysis retaining each dose as a separate node is reported in Supplementary Table S7. Outcomes reported only as medians without a measure of dispersion could not be converted to means and standard deviations and were not pooled.

A small number of arm-level values were recorded in the workbook as estimated rather than extracted. These are identified in Supplementary Table S8 and are the subject of a dedicated sensitivity analysis (Section 3.9).

### 2.4 Risk of bias assessment

The methodological quality of the included trials was assessed using the Cochrane Risk of Bias 2 (RoB 2) tool. Five domains were evaluated: randomisation process, deviations from intended interventions, missing outcome data, measurement of the outcome, and selection of the reported result. For crossover trials the corresponding RoB 2 extension was used.

### 2.5 Statistical analysis and data synthesis

Networks were fitted for each outcome separately using the graph-theoretical frequentist approach implemented in the netmeta package for R, under a random-effects model, with passive recovery as the reference. Standardised mean differences (SMD) with 95% confidence intervals were the effect measure. Outcomes were oriented so that a negative SMD favours the treatment. Trials that could not be connected to the reference through any path were excluded from the corresponding network; one trial (25), which compared two active-recovery protocols with no passive arm, was excluded from every network on this basis.

Statistical heterogeneity was quantified using tau-squared and the *I*-squared statistic with its 95% confidence interval, values above 50% indicating substantial heterogeneity; where a network contained no replicated design, heterogeneity was not estimable and this is reported as such. Inconsistency was assessed by node-splitting and by the design-by-treatment interaction test. Small-study effects were examined with comparison-adjusted funnel plots and Egger's regression test.

Treatment rankings are reported as *P*-scores. *P*-scores are presented only alongside the corresponding SMD and confidence interval, since a *P*-score expresses the probability of a treatment ranking best within a network and carries no information about whether any treatment is effective.

The certainty of evidence for each comparison against passive recovery was rated using CINeMA across six domains: within-study bias, reporting bias, indirectness, imprecision, heterogeneity and incoherence. A minimal important difference of SMD = 0.5 was specified for the imprecision, heterogeneity and incoherence domains.

*A priori* subgroup analyses were conducted by sport environment (summer vs. winter). Sensitivity analyses were performed by restricting to confirmed Tier 4–5 participants, excluding trials rated at some concerns for risk of bias, excluding the sham node, excluding adolescent cohorts, and excluding arm-level values recorded as estimated rather than extracted.

All analyses were performed in R (version 4.4.2) using the netmeta and ggplot2 packages. The analysis code and the arm-level dataset are available at (repository/on request) so that the analysis can be reproduced in full.

## 3 Results

### 3.1 Study selection

**Figure 1:**
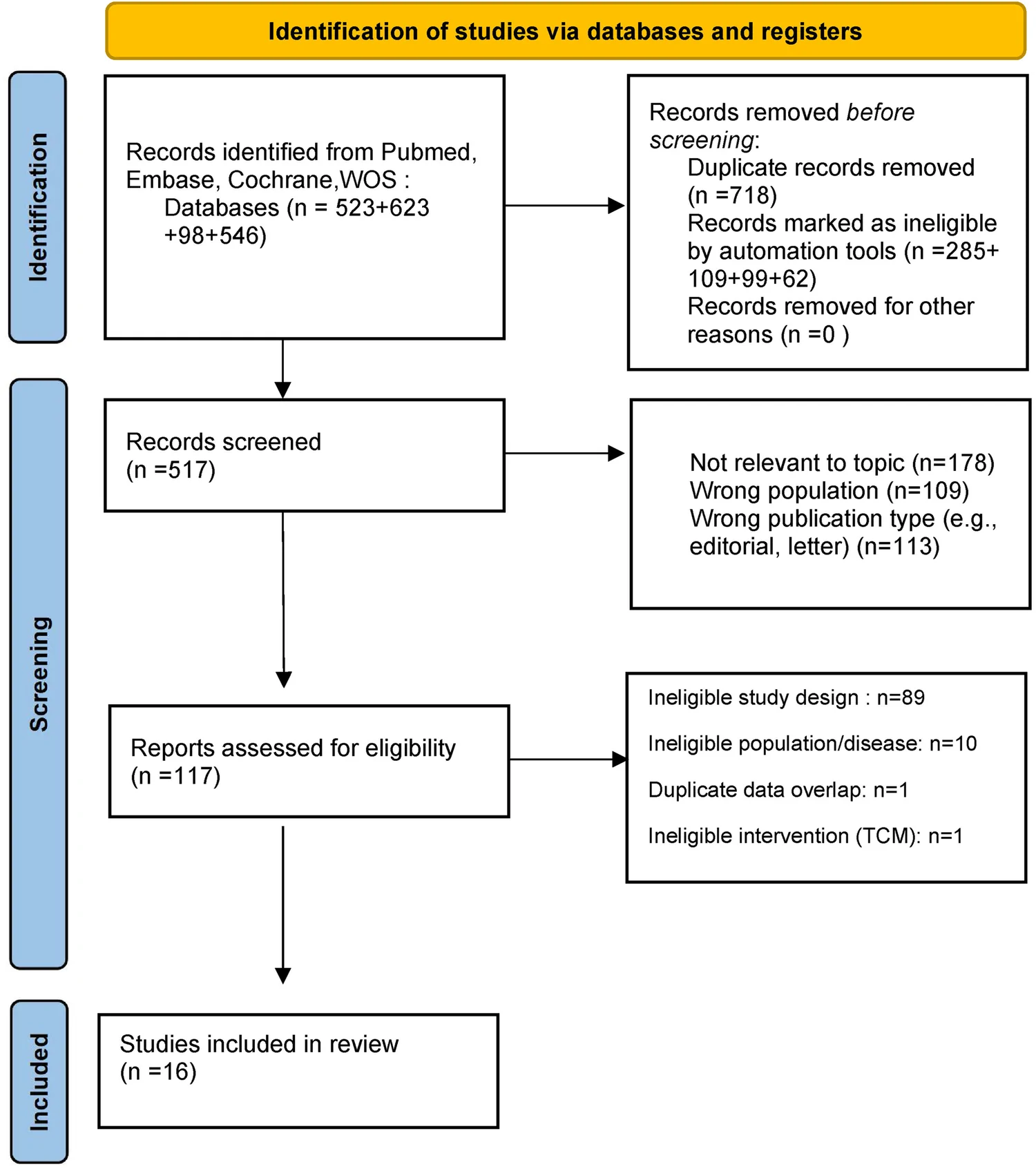
PRISMA flow diagram of the study selection process.

The systematic search of four databases yielded 1,790 records (PubMed 523, Embase 623, CENTRAL 98, Web of Science 546). After removal of 718 duplicates and exclusion of 555 records at automated screening, 517 abstracts were reviewed, of which 63 full texts were assessed. Sixteen randomised controlled trials met the inclusion criteria (2, 21–35) (Figure 1).

### 3.2 Study characteristics

The sixteen trials enrolled 273 participants across running, cycling, cross-country skiing, swimming and triathlon (Table 1). Training status was not uniform: cohorts were described by their original authors as elite or professional, highly trained, or competitive, and four cohorts were adolescents [Carvalho et al. (28), mean age 13.6 years; Batista et al. (22), 14.1 years; Tardieu-Berger et al. (25), 16.6 years; Neric et al. (31), 17.7 years]. Only seven trials reported VO2max, so eligibility against our stated criterion could not be verified for the remaining nine; of the seven, Overmayer et al. (32) reported values below that criterion (50 mL/kg/min in males, 46 in females). Full characteristics, including reported VO2max against the eligibility criterion, are given in Supplementary Table S5.

**Table 1:** Characteristics of the included studies.

Author (year)	Sport type	Participants (*N*, sex)	Age (years)	Training level	Intervention arms	Poolable outcomes
Li et al. (2025) (21)	Treadmill running	12 (M)	NR	Professional	Cooling blanket vs. natural cooling	None poolable (medians only)
Batista et al. (2024) (22)	Swimming	20 (12M/8F)	14.1	Competitive (adolescent)	CWI vs. thermoneutral immersion (sham) vs. PR	Flexibility, CMJ, Performance
Ali et al. (2011) (23)	Running (10-km TT)	12 (9M/3F)	NR	Competitive (VO2max 68.7)	CG (low/med/high) vs. PR	RPE, CMJ, performance
Argus et al. (2013) (2)	Track cycling	11 (M)	NR	Highly trained	CG, EMS, humidification vs. PR	BLA, performance
Bieuzen et al. (2014) (24)	Trail running	11 (M)	NR	Highly trained (VO2max 60.1)	CG (25 mmHg), CG (20 mmHg) vs. PR	DOMS, CK, RPE, IL-6, CMJ, performance
Halson et al. (2014) (26)	Cycling	21 (M)	NR	Competitive	CWI vs. PR	Performance
Govus et al. (2018) (27)	XC skiing	32 (18M/14F)	NR	Elite	CG, NMES vs. AR control	DOMS, CK, CMJ
Carvalho et al. (2023) (28)	Swimming	19 (11M/7F)	13.6	Competitive (adolescent)	SM, DM vs. PR	Flexibility, CMJ, performance
Losnegard et al. (2015) (29)	XC skiing	10 (M)	NR	Elite (VO2max 73.0)	AR vs. PR	BLA, performance
Marquet et al. (2015) (30)	BMX cycling	11 (7M/4F)	NR	Elite	CWI, AR, CHO drink vs. PR	DOMS, performance
Neric et al. (2009) (31)	Swimming	30 (19M/11F)	17.7	Competitive (adolescent)	AR, EMS vs. PR	BLA, performance
Overmayer et al. (2017) (32)	Track cycling	21 (13M/8F)	NR	Trained (VO2max 50 M/46 F)	IPC vs. PR	BLA, RPE, performance
Hausswirth et al. (2011) (33)	Trail running	9 (M)	NR	Highly trained (VO2max 62.0)	WBC vs. FIR vs. PR	DOMS, CK, CMJ, performance
Schaal et al. (2015) (34)	Sync. swimming	10 (F)	NR	Elite (VO2max 62.1)	WBC vs. PR	BLA, RPE, performance
Stearns et al. (2018) (35)	Triathlon	33 (22M/11F)	NR	Elite	CWI vs. PR	DOMS, CK, IL-6, RPE, performance
Tardieu-Berger et al. (2004) (25)	Running (intermittent)	11 (M)	16.6	Trained (adolescent)	Structured AR vs. standard AR	Not connected to PR

Rebuilt from the extraction workbook. AR, active recovery; CG, compression garments; CHO, carbohydrate; CK, creatine kinase; CMJ, countermovement jump; CWI, cold-water immersion; DM, deep massage; EMS/NMES, electrical stimulation; FIR, far-infrared; IPC, intermittent pneumatic compression; PR, passive recovery; SM, superficial massage; WBC, whole-body cryotherapy. The final column lists only outcomes with at least two arms carrying a numeric mean and standard deviation.

Ten modalities were represented. Two trials contributed arms outside the stated PICO—a carbohydrate drink (30) and humidification therapy (2)—and one contributed a thermoneutral-immersion sham (22). Tardieu-Berger et al. (25) compared two active-recovery protocols with no passive comparator and could not be connected to any network.

### 3.3 Risk of bias

Six of the sixteen trials were rated at overall low risk of bias; ten raised some concerns (Supplementary Figure S4). Concerns arose almost entirely in the domain of deviations from intended interventions (D2), since blinding of participants is not feasible for physical modalities. No trial raised concerns for missing outcome data (D3) or measurement of the outcome (D4).

### 3.4 Network geometry and the size of the evidence base

**Figure 2:**
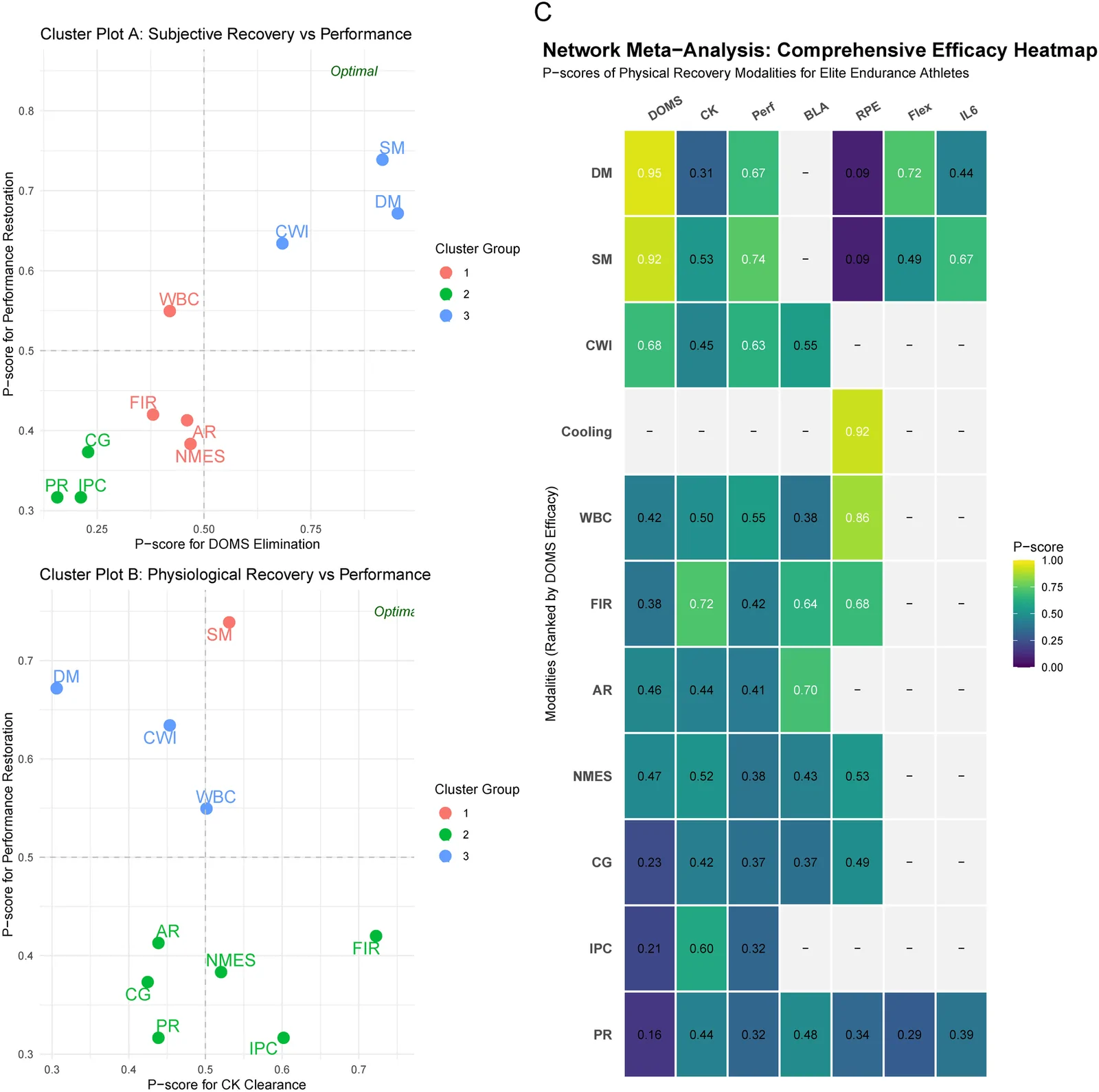
Network geometry for the three primary outcomes. **(A)** Delayed-onset muscle soreness; **(B)** creatine kinase; **(C)** sport-specific performance. Node size is proportional to the number of contributing trials and edge thickness to the number of direct comparisons. Massage does not appear in the soreness network and intermittent pneumatic compression does not appear in the creatine kinase network, because no trial provided the corresponding data.

The networks are considerably smaller than the count of sixteen trials suggests, because most trials measured only two or three of the eight outcomes. The DOMS network drew on 5 trials and 7 nodes; creatine kinase on 4 trials and 7 nodes; blood lactate on 5 and 7; RPE on 5 and 5; interleukin-6 on 2 and 3; flexibility on 2 and 5; countermovement jump or maximal voluntary contraction on 6 and 10; and sport-specific performance on 11 and 11 (Figure 2).

Across the eight networks, 35 of 55 nodes (64%) were informed by a single trial (Supplementary Table S4). Five of the seven nodes in the creatine kinase network, eight of the ten in the CMJ network and four of the five in the flexibility network rest on one trial each. Far-infrared therapy was a single-trial node in every network in which it appeared, as was intermittent pneumatic compression. Rankings derived from single-trial nodes are unstable by construction and are reported below only for completeness.

No massage arm carried a numeric DOMS or interleukin-6 value, and the only trial of intermittent pneumatic compression (32) did not measure creatine kinase; these modalities are therefore absent from the corresponding networks. The only cooling-blanket trial (21) reported RPE as medians without dispersion and could not be pooled.

### 3.5 Primary outcomes

#### 3.5.1 Delayed-onset muscle soreness

Five trials contributed (Figure 2A). No modality differed significantly from passive recovery. Point estimates ranged from SMD = −1.31 (95% CI −3.45 to 0.84) for active recovery to −0.56 (−3.31 to 2.19) for far-infrared therapy, with every interval spanning roughly four SMD units. Heterogeneity was extreme (*I*-squared 90.2%, 95% CI 73.9–96.3; tau-squared 1.74; tau 1.32; *Q* = 20.39, df = 2, *P* < 0.001). A between-study standard deviation of true effects of 1.32 SMD units exceeds the magnitude of any individual point estimate in the network. This network is also acutely unstable: excluding two trials whose values were recorded as estimated rather than extracted reverses the conclusion entirely (Section 3.9). We do not regard the pooled DOMS estimates as interpretable in either direction.

#### 3.5.2 Creatine kinase

**Figure 3:**
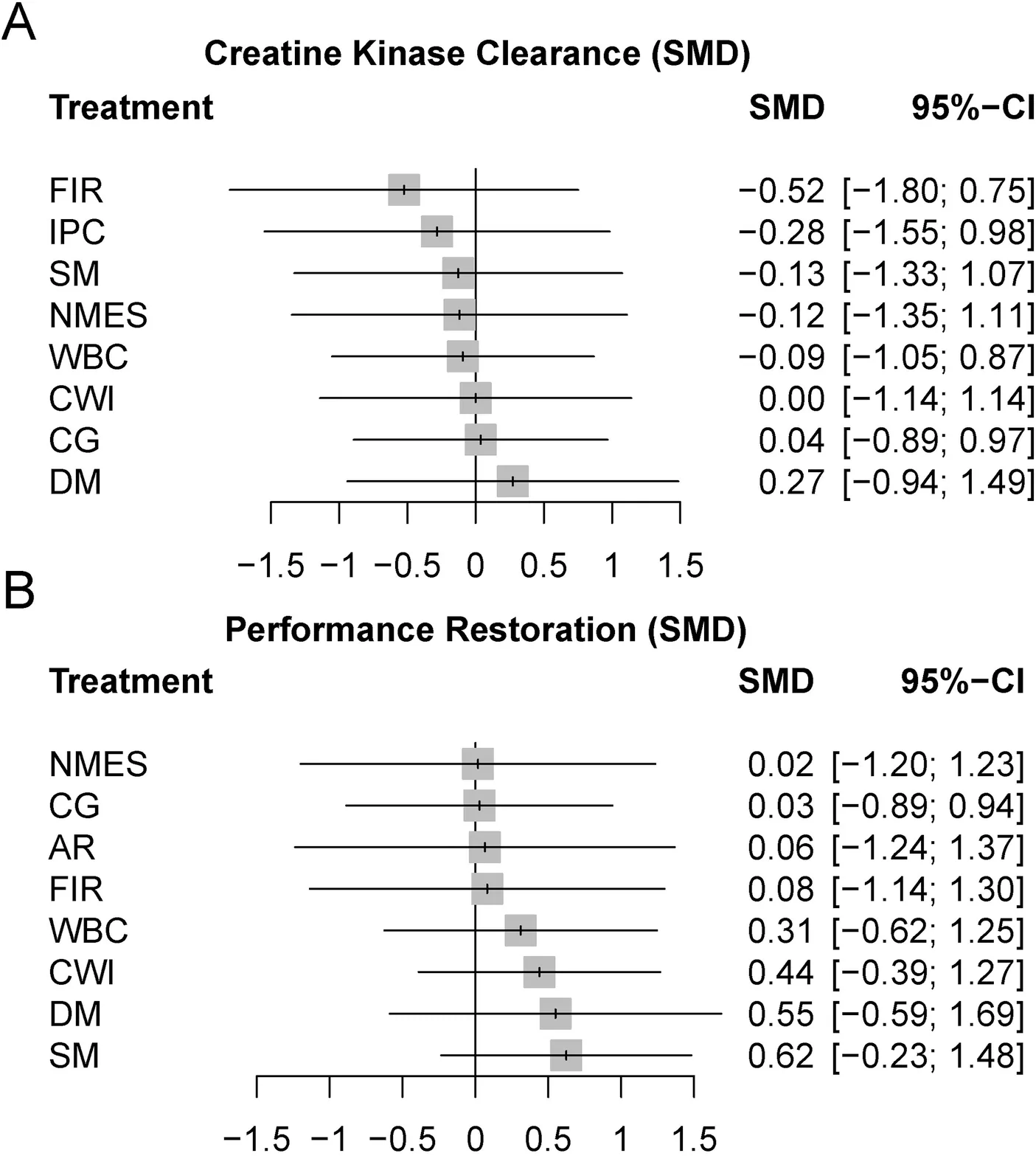
Forest plots of each modality against passive recovery. **(A)** Creatine kinase; **(B)** sport-specific performance. A negative SMD favours the treatment. No contrast in any of the three networks excludes the null.

Four trials contributed (Figure 3A). No modality differed significantly from passive recovery; far-infrared therapy ranked third (SMD = −0.25, 95% CI −1.17 to 0.68; *P*-score 0.651). This network contains no closed loop and no replicated design, so its between-study variance could not be estimated (df = 0) and the random-effects model reduces to a common-effect model. The confidence intervals reported for this outcome therefore incorporate no between-study heterogeneity and should be read as narrower than the evidence warrants, not wider. The same applies to the interleukin-6 and flexibility networks.

#### 3.5.3 Sport-specific performance

Eleven trials contributed (Figure 3B), making this the best-supported network. No modality differed significantly from passive recovery. Whole-body cryotherapy ranked highest (SMD = −0.71, 95% CI −1.62 to 0.19; *P*-score 0.879) on the basis of a single trial; cold-water immersion, with three trials, gave SMD = −0.27 (−0.70 to 0.16). Heterogeneity was absent (*I*-squared 0%, 95% CI 0–67.6; *Q* = 1.5, df = 7, *P* = 0.98). This is the one network in which the absence of an effect is informative rather than merely uninformative, and we return to it in the Discussion.

### 3.6 Secondary outcomes

#### 3.6.1 Blood lactate

Five trials contributed. Passive recovery outranked active recovery (*P*-scores 0.620 and 0.482); the active-recovery point estimate favoured the control (SMD = +0.22, 95% CI −0.74 to 1.19). Whole-body cryotherapy was significantly worse than passive recovery (SMD = +2.50, 95% CI 0.81–4.19; *P* = 0.004) on the basis of one trial (34). We do not interpret this as harm: the network pools three different constructs—lactate clearance (31, 32), peak lactate (25) and post-time-trial lactate (34)—and the cryotherapy estimate derives from the last, where a higher post-trial concentration may reflect greater work performed rather than poorer recovery. Heterogeneity was substantial (*I*-squared 74.5%, 95% CI 15.2–92.3; tau-squared 0.39).

#### 3.6.2 Rating of perceived exertion

Five trials, five nodes. No modality differed significantly from passive recovery. Cold-water immersion ranked highest (SMD = −0.50, 95% CI −1.22 to 0.22; *P*-score 0.872) from a single trial. Whole-body cryotherapy and intermittent pneumatic compression both gave point estimates of exactly 0.00. Heterogeneity was absent (*I*-squared 0%; *Q* = 0.1, df = 1, *P* = 0.75).

#### 3.6.3 Interleukin-6 and flexibility

Two trials each. Nothing approached significance; the largest estimate was SMD = −0.27 for the sham immersion on flexibility. Both networks have df = 0 (see Section 3.5).

#### 3.6.4 Countermovement jump and maximal voluntary contraction

Six trials, ten nodes, of which eight rest on a single trial. Two contrasts excluded the null: whole-body cryotherapy (SMD = −1.55, 95% CI −2.80 to −0.30; *P* = 0.015) and compression garments (SMD = −0.77, 95% CI −1.49 to −0.04; *P* = 0.039). The cryotherapy estimate comes from one trial. This network pools absolute jump height, change scores and percentage of pre-test MVC; we regard both contrasts as artefacts of that pooling and draw no inference from them (*I*-squared 51.1%, 95% CI 0–87.6).

### 3.7 Sham comparison

Batista et al. (22) included a thermoneutral-immersion (27 degrees C) arm intended as a sham. Entered as its own node, it ranked highest in the flexibility network (*P*-score 0.683, above cold-water immersion at 0.516, superficial massage at 0.509 and deep massage at 0.439), third of eleven nodes for sport-specific performance (0.671, above deep massage at 0.487, superficial massage at 0.383, active recovery at 0.395 and compression garments at 0.333), and above both massage arms for countermovement jump (0.372 against 0.288 and 0.283). No sham contrast reached significance, and all derive from one trial, so no inference about thermoneutral immersion itself is warranted. The consistency of the pattern across three outcomes is nonetheless what an expectancy account would predict, and we return to it in the Discussion.

### 3.8 Heterogeneity and inconsistency

Heterogeneity statistics for every network are reported in Supplementary Table S3 and the design-based *Q* decomposition in Supplementary Table S3b. Three networks (creatine kinase, interleukin-6, flexibility) have zero degrees of freedom and no estimable heterogeneity. Of the remainder, DOMS (*I*-squared 90.2%) and blood lactate (74.5%) exceed the pre-specified 50% threshold, CMJ sits at 51.1%, and RPE and performance show none. Node-splitting detected no significant inconsistency between direct and indirect evidence (all *P* > 0.05; Supplementary Figure S3), but with so few closed loops this test has minimal power and its result is not evidence of consistency. Comparison-adjusted funnel plots (Supplementary Figure S5) were visually symmetrical; with sixteen trials spread across these networks the assessment of small-study effects is underpowered and we regard it as inconclusive.

### 3.9 Sensitivity analyses

Six scenarios were examined (Supplementary Table S7). The dominant finding is not that the results were robust, but that most scenarios could not be fitted at all.

Restricting the analysis to participants of confirmed Tier 4–5 status left no estimable network for creatine kinase, countermovement jump, interleukin-6 or flexibility, and reduced sport-specific performance to three trials, DOMS to three, and blood lactate and RPE to two each. Excluding trials rated at some concerns for risk of bias left no network for blood lactate, interleukin-6 or flexibility, and reduced DOMS and creatine kinase to two trials each. Excluding adolescent cohorts removed the flexibility network. That this evidence base largely cannot survive the application of its own eligibility criterion is, in our view, among the more informative results of this review.

Removing the sham node changed nothing. Excluding trials at some concerns for risk of bias, and excluding adolescent cohorts, left the three contrasts described in Sections 3.5 and 3.6 unchanged in direction and magnitude.

#### 3.9.1 One scenario changed the results completely

When arm-level values recorded in the workbook as estimated rather than extracted were excluded, the DOMS network fell from five trials to three and four modalities became significantly superior to passive recovery: active recovery (SMD = −2.65, 95% CI −3.71 to −1.60), compression garments (−2.80, −4.16 to −1.44), cold-water immersion (−2.51, −3.54 to −1.47) and neuromuscular electrical stimulation (−2.99, −4.35 to −1.63).

This is not evidence that these modalities work. The two excluded trials (24, 35) are precisely the two whose DOMS arms are numerically identical (3.0, 3.0 and 3.0; 60.0 and 60.0), yielding standardised mean differences of exactly zero, and both sets of values are recorded as estimated rather than extracted. Removing them leaves a network in which one trial (30) carries almost all the weight: its passive arm reported a DOMS score of 5.3 with a standard deviation of 0.5, against approximately 3.5 in each of its three other arms, and that small standard deviation gives the contrast a very large weight. The apparent effects for compression garments and electrical stimulation are inherited indirectly from that single passive-recovery contrast through (27).

Neither version of the DOMS network can be trusted. The null result of the main analysis rests on two trials whose values were not extracted from the source publications, and the large effects of the sensitivity analysis rest on one trial with an anomalous control arm. We report both and conclude from neither. Resolving the estimated values against the source publications is the first priority for any future analysis of this outcome: it determines whether the network shows nothing or an effect of nearly three standard deviations.

### 3.10 Certainty of evidence

Certainty was rated for all 47 comparisons against passive recovery (Supplementary Table S6). Thirty-one were rated very low and fifteen low. Imprecision was the dominant reason for downgrading, rated as major concerns for 32 of 47 comparisons; incoherence contributed substantially, since the creatine kinase, interleukin-6 and flexibility networks contain no closed loop and the design-by-treatment interaction test is not estimable in them. Reporting bias was rated as suspected throughout, because with sixteen trials distributed across these networks the funnel plots cannot exclude small-study effects. Every comparison in the creatine kinase, interleukin-6 and flexibility networks was rated very low.

One comparison was rated moderate: compression garments vs. passive recovery for sport-specific performance (SMD = 0.04, 95% CI −0.37 to 0.45), rated as no concerns on within-study bias, indirectness, imprecision, heterogeneity and incoherence, and downgraded once only for suspected reporting bias. The single most certain result in this review is that compression garments do not improve sport-specific performance in endurance athletes.

**Figure 4:**
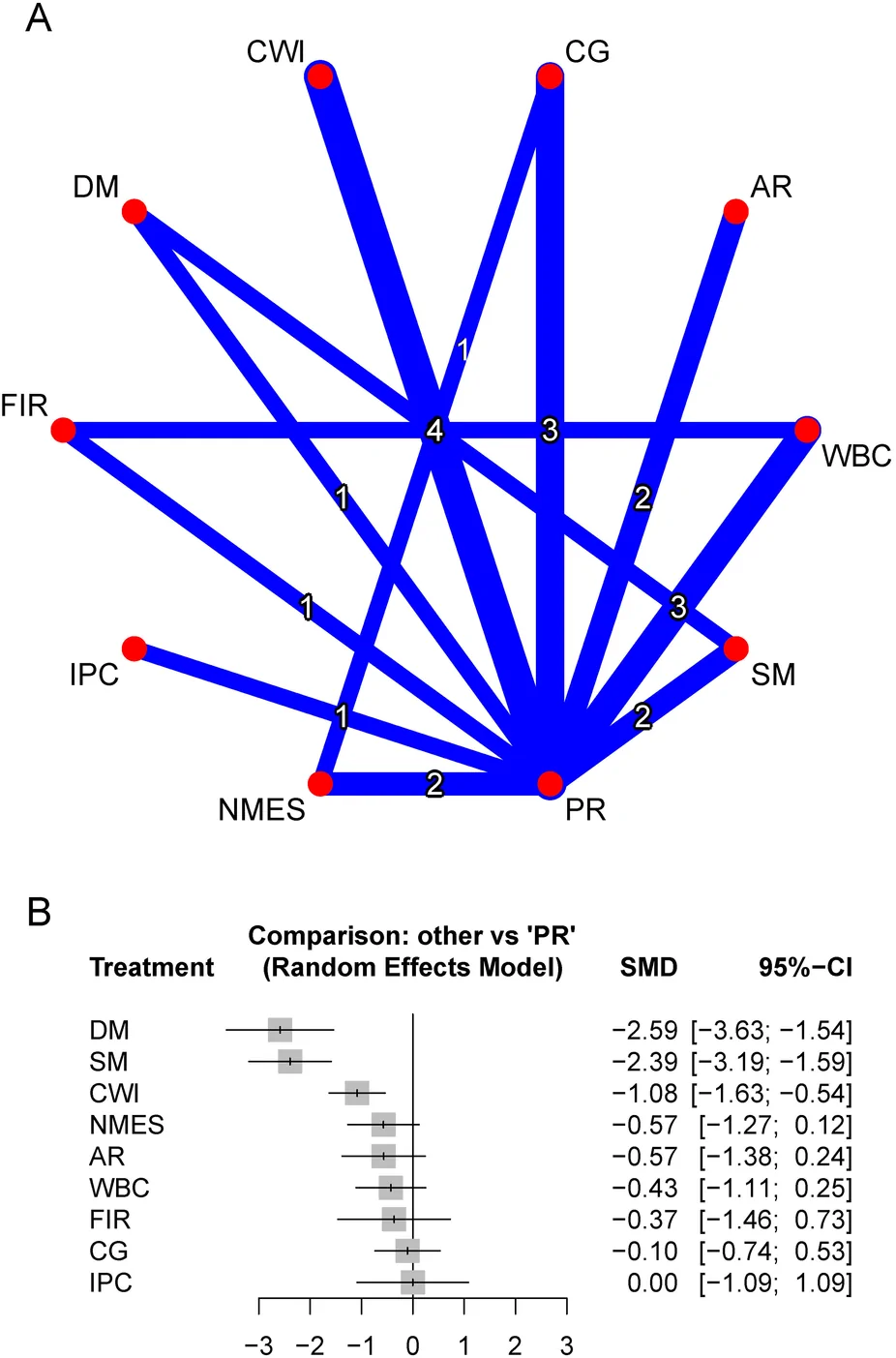
**(A)** *P*-scores across all eight outcomes. A *P*-score is the probability of a treatment ranking best within its network and is not evidence of efficacy. **(B)** Certainty of evidence for each comparison against passive recovery, assessed with CINeMA.

Two further performance comparisons—active recovery (SMD = −0.01, 95% CI −0.39 to 0.36) and neuromuscular electrical stimulation (SMD = 0.00, 95% CI −0.40 to 0.40)—were rated as no concerns for imprecision, their confidence intervals excluding clinically important effects in both directions, and were rated low overall (Figure 4B).

## 4 Discussion

### 4.1 Principal findings

Across eight outcomes and 47 comparisons, no physical recovery modality was shown to outperform passive recovery in competitive or elite endurance athletes. Three confidence intervals excluded the null, and none survives scrutiny: two arise from single-trial nodes, and all three come from networks in which incommensurable measures were pooled. Certainty of evidence was very low for two thirds of comparisons and never exceeded moderate.

This differs from what this literature usually reports, and it rests on four observations that we consider more informative than any ranking we could offer.

### 4.2 For performance, the null is informative

The distinction between “no evidence of an effect” and “evidence of no clinically important effect” is the one that matters for practice, and the two are not interchangeable. For sport-specific performance our data support the second, at least for three modalities. Compression garments (SMD = 0.04, 95% CI −0.37 to 0.45), active recovery (−0.01, −0.39 to 0.36) and neuromuscular electrical stimulation (0.00, −0.40 to 0.40) all have intervals lying entirely within the range of effects we pre-specified as clinically unimportant. Heterogeneity in this network was absent (*I*-squared 0%), and it is the best-supported network in the review, drawing on eleven trials. The compression garment comparison is the only one in the entire review rated at moderate certainty.

For an athlete or coach this is actionable: whatever else compression garments may do, the randomised evidence indicates with reasonable confidence that they do not make the next performance better. Elsewhere in the review the null results are uninformative, and we do not present them otherwise.

### 4.3 The evidence base is thinner than it appears

Sixteen randomised trials sounds like a body of evidence. It is not. Most trials measured two or three of the eight outcomes, so the networks are far smaller than the trial count implies: the DOMS network draws on five trials, creatine kinase on four, interleukin-6 and flexibility on two each. Thirty-five of the 55 nodes across the eight networks (64%) are informed by a single trial. Far-infrared therapy and intermittent pneumatic compression are single-trial nodes in every network in which they appear.

The consequences are structural rather than incidental. In the DOMS network, heterogeneity reached *I*-squared 90.2%, with a between-study standard deviation of true effects of 1.32 SMD units—larger than any point estimate in that network. Pooling under those conditions produces intervals four SMD units wide, which cannot discriminate between a large benefit and a large harm. In the creatine kinase, interleukin-6 and flexibility networks the opposite problem arises: with no replicated design, between-study variance cannot be estimated at all, the random-effects model collapses to a common-effect model, and the reported intervals are narrower than the evidence justifies.

The clearest demonstration is our own sensitivity analysis. Removing two of the five DOMS trials converts a network in which nothing is significant into one in which four modalities are superior by roughly three standard deviations (Section 3.9). A body of evidence in which the answer depends on which two of five trials are included is not a body of evidence from which recommendations can be drawn. We think this deserves emphasis because it is invisible in the way such reviews are usually summarised: a ranking table with ten modalities implies ten bodies of evidence, where here it represents, for several modalities, one small trial each.

The point is reinforced by what happens when the review's own eligibility criterion is enforced. Restricting to participants of confirmed elite status leaves four of the eight outcomes with no estimable network at all, and the best-supported remaining outcome with three trials. The elite-specific evidence for physical recovery modalities, as opposed to the evidence borrowed from mixed and adolescent cohorts, is close to absent.

### 4.4 A sham performed as well as the interventions

One included trial (22) employed a thermoneutral-immersion (27 degrees C) sham. Entered as its own node, it ranked highest in the flexibility network, third of eleven for sport-specific performance, and above both massage arms for countermovement jump (Section 3.7).

No sham contrast reached significance and all derive from a single trial, so we draw no inference about thermoneutral immersion itself. What is notable is the consistency of the pattern across three outcomes, and its direction: the sham was not merely non-inferior to the active modalities, it out-ranked most of them. This is what an expectancy account of this literature predicts, and it is difficult to reconcile with an account in which the active modalities exert modality-specific physiological effects of the size usually claimed.

The point generalises. Physical recovery modalities cannot be blinded; the athlete always knows. The outcomes reported most often—soreness and perceived exertion—are those most sensitive to expectation. Across this literature the modalities with the most salient sensory experience tend to rank best on precisely those outcomes. That pattern is consistent with a real effect and equally consistent with an expectancy effect, and the trials as designed cannot distinguish them. Until sham-controlled trials are conducted, they will remain indistinguishable.

### 4.5 Relation to previous reviews

Previous meta-analyses have reported benefits of massage for soreness and fatigue (36), of cold-water immersion for recovery from strenuous exercise (37), and of whole-body cryotherapy for muscle soreness (38). Our findings do not contradict the underlying trials so much as the confidence usually placed in them. Three differences in approach account for most of the divergence: we restricted inclusion to competitive endurance athletes, in whom the therapeutic window may be narrower; we required arm-level data with a numeric measure of dispersion, which excluded several frequently cited results; and we report certainty of evidence, which no previous review in this area has done. Where our estimates disagree in direction with earlier work, they do so within intervals wide enough to contain both.

Notably, we did not reproduce the finding that active recovery is the reference strategy for lactate clearance: in our network, passive recovery out-ranked it (*P*-scores 0.62 against 0.48). We do not present this as evidence against active recovery. The lactate network pools three constructs—clearance, peak concentration and post-time-trial concentration—and the comparison is not well posed until those are separated.

### 4.6 Limitations

The limitations of this review are largely the limitations of the evidence it summarises, but three are our own.

First, this analysis is a correction of our own earlier work. In the version of this manuscript originally submitted, four trials had been coded as delivering interventions they did not deliver, and four reported results had no corresponding data in our extraction workbook. Those errors were identified during peer review and are documented in full in our response to the reviewers and in Supplementary Table S8. The present analysis has been re-coded from the extraction workbook and re-run in its entirety; the analysis code and the arm-level dataset are available so that it can be checked.

Second, a small number of arm-level values in our workbook were recorded as estimated rather than extracted, and Section 3.9 shows that the DOMS result depends entirely on them. We have not resolved these against the source publications and we do not present a DOMS conclusion in either direction until they are.

Third, several outcome nodes pool measures that are not commensurable. The blood-lactate node combines clearance, peak and post-trial concentrations; the countermovement jump node combines absolute jump height, change scores and percentage of pre-test maximal voluntary contraction. All three of the confidence intervals in this review that exclude the null arise in these two networks, and we regard them as artefacts of that pooling rather than findings.

Beyond these, training status was heterogeneous: only seven of sixteen trials reported VO2max, one of the seven fell below our stated criterion, and four cohorts were adolescents. The review also inherits the sparsity, the unblinded designs and the absence of sham controls described above. The funnel plots were symmetrical, but with sixteen trials across these networks that assessment is underpowered and we do not treat it as reassuring.

### 4.7 Implications

#### 4.7.1 For practice

The randomised evidence does not support recommending one physical recovery modality over another on grounds of comparative efficacy, and for compression garments it indicates with moderate certainty that no performance benefit should be expected. This does not mean the modalities are useless; it means the justification for them cannot presently be efficacy on these outcomes. Athlete preference, perceived benefit, cost and opportunity cost are legitimate grounds for a decision, and given the sham result they may be the honest ones.

#### 4.7.2 For research

Further small unblinded trials of single modalities against passive rest will not resolve this. What is needed is a smaller number of adequately powered trials with sham or attention-control comparators, pre-registered outcomes, and a commitment to reporting arm-level means and standard deviations so that the results can enter synthesis. A construct-specific definition of the lactate and neuromuscular outcomes would also be valuable; at present these labels cover measures that answer different questions.

## 5 Conclusion

In competitive and elite endurance athletes, no physical recovery modality has been shown to outperform passive recovery on any outcome we examined. For sport-specific performance the evidence goes further: compression garments, active recovery and neuromuscular electrical stimulation can be said, with low to moderate certainty, not to produce a clinically important benefit. Elsewhere the randomised base is too small and too heterogeneous to support the modality-specific recommendations routinely made in elite sport—the muscle soreness network reverses its conclusion when two of its five trials are removed—and a sham immersion performed at least as well as the active modalities on every outcome to which it contributed. The comparative efficacy of physical recovery modalities in endurance athletes remains, on the present evidence, undemonstrated.

## Data Availability

The original contributions presented in the study are included in the article/Supplementary Material, further inquiries can be directed to the corresponding authors.
