# Supplementary material for: Physical recovery modalities in competitive and elite endurance athletes: a systematic review and network meta-analysis finding limited evidence of benefit over passive recovery
Source: Front Sports Act Living. 2026 Aug 18;8:1876924. doi: 10.3389/fspor.2026.1876924 (PMC13531764; doi:10.3389/fspor.2026.1876924)
Supplement: Supplementary file 1 [file Datasheet1.docx]

**Supplementary Figure S1. Forest plots for secondary recovery outcomes.**

Summary of relative effects compared to passive recovery (PR) for: (A) Blood Lactate (BLA) clearance rate; (B) Rating of Perceived Exertion (RPE); (C) Flexibility (Sit-and-reach performance); and (D) Interleukin-6 (IL-6) inflammatory marker. All estimates are expressed as Standardized Mean Differences (SMD).

**Supplementary Figure S2. Subgroup analyses of recovery efficacy based on sport environment and measurement timing.** This figure illustrates how the relative effectiveness of recovery modalities is modified by external factors.(A) Comparison of efficacy between Summer and Winter endurance sports.(B) Time-course evolution of recovery effectiveness across different follow-up intervals.

**Supplementary Figure S3. Node-splitting plots for inconsistency assessment.**

Visual representation of the consistency between direct and indirect evidence for all closed loops in the network. A p-value > 0.05 indicates no significant inconsistency, supporting the validity of the network meta-analysis results.

**Supplementary Figure S4. Risk of bias assessment (Traffic light plot).**

Assessment of individual studies using the Cochrane Risk of Bias 2 (RoB 2) tool. Domains include: D1 (Randomization process), D2 (Deviations from intended interventions), D3 (Missing outcome data), D4 (Measurement of the outcome), and D5 (Selection of the reported result). Green indicates low risk, and yellow indicates some concerns.

**Supplementary Figure S5. Comparison-adjusted funnel plots for publication bias.**

Funnel plots for the primary outcomes (DOMS and Performance). The symmetry of the plots was used to assess the potential presence of small-study effects or publication bias across the network.

| DM | 0.12 (-0.3, 0.5) | 0.08 (-0.4, 0.6) | 0.45 (0.1, 0.8)* | 0.88 (0.4, 1.3)* |
| --- | --- | --- | --- | --- |
| 0.05 (-0.2, 0.3) | SM | 0.04 (-0.3, 0.4) | 0.41 (0.0, 0.8)* | 0.92 (0.5, 1.4)* |
| *0.25 (0.0, 0.5) ** | 0.20 (-0.1, 0.4) | CWI | 0.35 (-0.1, 0.7) | 0.75 (0.3, 1.2)* |
| *0.55 (0.2, 0.9) ** | *0.50 (0.1, 0.8) ** | *0.30 (0.0, 0.6) ** | WBC | 0.38 (-0.1, 0.8) |
| *0.85 (0.5, 1.2) ** | *0.80 (0.4, 1.1) ** | *0.60 (0.3, 0.9) ** | 0.32 (-0.1, 0.7) | PR |

**Table S1. League Table of Relative Effects for Performance and DOMS**

Legend S1: *Upper-right triangle: Performance restoration; Lower-left triangle: DOMS elimination. Values are Standardized Mean Differences (SMD) with 95% Confidence Intervals. Significant difference (p < 0.05).

| Step | Search Terms (PubMed / MEDLINE) |
| --- | --- |
| #1 (P) | Endurance athletes[Mesh] OR "elite athletes"[tw] OR "professional athletes"[tw] OR "highly-trained"[tw] OR "competitive athletes"[tw] OR "runners"[tw] OR "cyclists"[tw] OR "swimmers"[tw] OR "cross-country skiers"[tw] OR "triathletes"[tw] OR "marathoners"[tw] |
| #2 (I) | Physical therapy modalities[Mesh] OR "recovery modalities"[tw] OR "cold water immersion"[tw] OR "cryotherapy"[tw] OR "massage"[tw] OR "foam rolling"[tw] OR "compression garments"[tw] OR "active recovery"[tw] OR "electrostimulation"[tw] OR "pneumatic compression"[tw] OR "far-infrared"[tw] OR "hydrotherapy"[tw] |
| #3 (O) | Fatigue[Mesh] OR "muscle soreness"[tw] OR "DOMS"[tw] OR "creatine kinase"[tw] OR "blood lactate"[tw] OR "performance restoration"[tw] OR "muscle damage"[tw] OR "exercise recovery"[tw] |
| #4 (S) | Randomized Controlled Trial[pt] OR "randomized"[tw] OR "placebo"[tw] OR "randomly"[tw] OR "trial"[tw] OR "groups"[tw] OR "crossover"[tw] |
| #5 | #1 AND #2 AND #3 AND #4 |

**Table S2. Search strategy for PubMed**
